# Validation of Automatically Quantified Swim Stroke Mechanics Using an Inertial Measurement Unit in Paralympic Athletes

**DOI:** 10.3390/bioengineering11010015

**Published:** 2023-12-23

**Authors:** Matthew Slopecki, Mathieu Charbonneau, Jean-Michel Lavallière, Julie N. Côté, Julien Clément

**Affiliations:** 1 Department of Kinesiology and Physical Education, McGill University, Montréal, QC H2W 1S4, Canada; julie.cote2@mcgill.ca (J.N.C.); jclement@insquebec.org (J.C.); 2 Institut National du Sport du Québec, Montréal, QC H1V 3N7, Canada; mcharbonneau@insquebec.org; 3 Swimming Natation Canada, Ottawa, ON K2P 0P7, Canada; jlavalliere@swimming.ca; 4 École de Technologie Supérieure, Montréal, QC H3C 1K3, Canada

**Keywords:** swimming, paralympic, workload monitoring, wearable, stroke measurement

## Abstract

Biomechanics and training load monitoring are important for performance evaluation and injury prevention in elite swimming. Monitoring of performance and swim stroke parameters is possible with inertial measurement units (IMU) but has not been validated in para-swimmers. The purpose of this study was to validate a single IMU-based system to accurately estimate pool-swam lap time, stroke count (SC), stroke duration, instantaneous stroke rate (ISR), and distance per stroke (DPS). Eight Paralympic athletes completed 4 × 50 m swims with an IMU worn on the sacrum. Strokes cycles were identified using a zero-crossing algorithm on the medio-lateral (freestyle and backstroke) or forward-backward (butterfly and breaststroke) instantaneous velocity data. Video-derived metrics were estimated using Dartfish and Kinovea. Agreement analyses, including Bland–Altman and Intraclass Correlation Coefficient (ICC), were performed on all outcome variables. SC Bland–Altman bias was 0.13 strokes, and ICC was 0.97. ISR Bland–Altman biases were within 1.5 strokes/min, and ICCs ranged from 0.26 to 0.96. DPS Bland–Altman biases were within 0.20 m, and ICCs ranged from 0.39 to 0.93. A single-IMU system can provide highly valid performance and swim stroke monitoring data for elite para-swimmers for the majority of strokes, with the exception of backstroke. Future work should improve bilateral stroke detection algorithms in this population.

## 1. Introduction

Musculoskeletal disorders and pain are commonly reported in swimming and para-swimming, but few validated tools exist to help guide their use from an injury prevention perspective [1,2]. For instance, such tools can theoretically help monitor the number of strokes performed and time spent swimming in a training session, and subsequently the overall training workload. Such tools could also help us to better understand swimming technique, reducing injury risk from poor technique performed over a chronic period. In the field, swim coaches identify swimming kinematic parameters as highly important to monitor [3]. Furthermore, the previous literature has shown that some mechanical variables of swim strokes have been linked to performance in swimming [4]. Specifically, stroke rate (SR) and stroke length (SL) have significant relationships to swimming velocity for all strokes (for SR) and for freestyle and butterfly (for SL) in elite swimmers [5]. In addition, automatic estimation of stroke count (SC) would allow for estimates of the number of shoulder rotations performed in training sessions. Monitoring the SC is of high importance due to the association between high workloads and shoulder pain in swimmers.

Ninety percent of young, elite swimmers have self-reported shoulder pain [1]. In addition, in a study of Polish national team para-swimmers, it was reported that 29% of them had shoulder pain in the 7 days prior to filling out a cross-sectional questionnaire [2]. The average competitive swimmer completes 30,000 rotations of each shoulder every week [6]. It is proposed that numerous shoulder movement repetitions can contribute to joint inflammation and pain [7]. As such, monitoring swimming time and the number of movement repetitions completed while swimming may help to prevent overuse injuries to the shoulder. Additionally, monitoring swim stroke technique may be useful in predicting injury risk longitudinally and adjusting higher-risk movement strategies. However, for this to happen, valid swim stroke detection tools must first be developed.

While two-dimensional video analysis has historically been the gold standard for performance quantification and monitoring in swimming [3,8,9], it presents many drawbacks, such as parallax errors, water turbulence [10], and time-consuming data processing [9,11]. An alternate solution with quick data processing times, such as an inertial measurement unit (IMU), would benefit high-performance teams [4,8,10,12]. IMUs are small, inexpensive wearable devices that can monitor instantaneous velocity [12,13,14] and kinematic parameters of the swim stroke [15,16,17,18]. IMU placement on the sacrum has been suggested to minimize the effect of drag and allow for good indications of swimmers’ instantaneous velocity [14], SC [13], instantaneous stroke rate (ISR) [15], and distance per stroke (DPS) [19]. In addition, IMU-based velocity metrics in the wall push-off, glide, stroke preparation, and free-swimming phases have been shown to help predict competitive swimmers’ progression of freestyle lap time over a 10-week period [20], highlighting IMUs as a viable tool to help improve technique and subsequently performance in able-bodied swimmers. However, lap times, SC, stroke duration, and ISR derived from sacrum-worn IMUs have only been validated in able-bodied swimmers. Furthermore, ISR was only validated in freestyle swimming [15], and DPS has not been validated in any population to date. This is a step that must be completed to ensure accurate monitoring of swimming performance and workload for all swim strokes in all categories of swimmers alike.

IMUs are an under-researched technology in para-swimming. Previous investigations have focused on automation detection of kicking mechanics [21,22], upper limb coordination [23], and joint kinematic changes following electrical stimulation [24]. However, all of this research uses either multiple sensors or has specific use cases. One previous study has shown that a single IMU placed on the sacrum is a valid solution for performance quantification in swimmers with impairments compared to gold-standard tether units. Instantaneous velocity estimates in the four swimming strokes of para-swimmers showed small Bland–Altman biases of 0.03–0.06 m.s^−1^, root mean square errors (RMSE) of 0.14–0.39 m.s^−1^, and ICC between 0.49–0.94 for all stroke types [14]. Fleiss [25] proposed that ICC values less than 0.40 were considered poor; 0.40 to 0.59 as fair; 0.60 to 0.74 as good; and 0.75 to 1.00 as excellent. As such, instantaneous velocity can be estimated using IMUs with small biases, low RMSE, and fair to excellent ICC values in swimmers with impairments [14]. However, a gap exists to study the automatic quantification of swim stroke mechanics in para-swimming using a single IMU. Specifically, no research to date has validated a single sacrum-worn IMU to quantify SC, stroke duration, ISR, DPS, and lap time in para-swimmers for all swimming strokes. The aim of the current study was to validate an IMU solution against the gold-standard two-dimensional video to quantify these variables in Paralympic swimmers completing the four swimming strokes.

## 2. Materials and Methods

### 2.1. Participants

Eight Paralympic athletes (4m/4f; age: overall = 23.25 years ± 2.87, m = 23.75 years ± 2.63, f = 22.00 years ± 3.16; height: overall = 158.74 cm ± 26.01, m = 172.83 cm ± 14.63, f = 144.65 cm ± 28.90; body mass: overall = 61.54 kg ± 17.00, m = 72.43 kg ± 14.91, f = 50.65 ± 11.65) participated in the current study. All athletes were classified by the World Para Swimming Classification Panels and compete at an international level. Details of participants’ disabilities, para-swimming classes, and strokes performed in the present study can be found in Table 1. The 8 recruited athletes represent the entire population of the Centre de Haute Performance, a national training center for para swimming. As such, this represents the entire population available to sample and exceeds the sample size used in previous para-swimming research using a sacrum-worn IMU [14]. Informed consent was obtained from all subjects involved in this study. This study was conducted according to the guidelines of the Declaration of Helsinki and approved by the Institutional Review Boards of McGill University (protocol code 22-05-021, approved on 03/10/2022) and École de Technologie Supérieure (protocol code H20221001, approved on 03/11/2022).

### 2.2. Instrumentation

**Figure 1:**
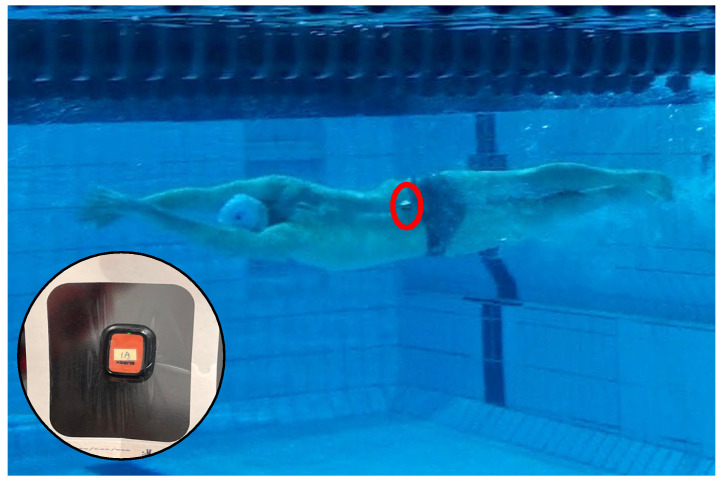
Placement of the IMU on the sacrum of an athlete.

A single IMU (±16 g, 120 Hz, Xsens Dot, Xsens Technologies, Enschede, The Netherlands) was placed between the participant’s posterior superior iliac spines (Figure 1). The IMU had dimensions of 36 × 30 × 11 mm and a mass of 3.15 g. Data were recorded on the IMU onboard memory.

**Figure 2:**
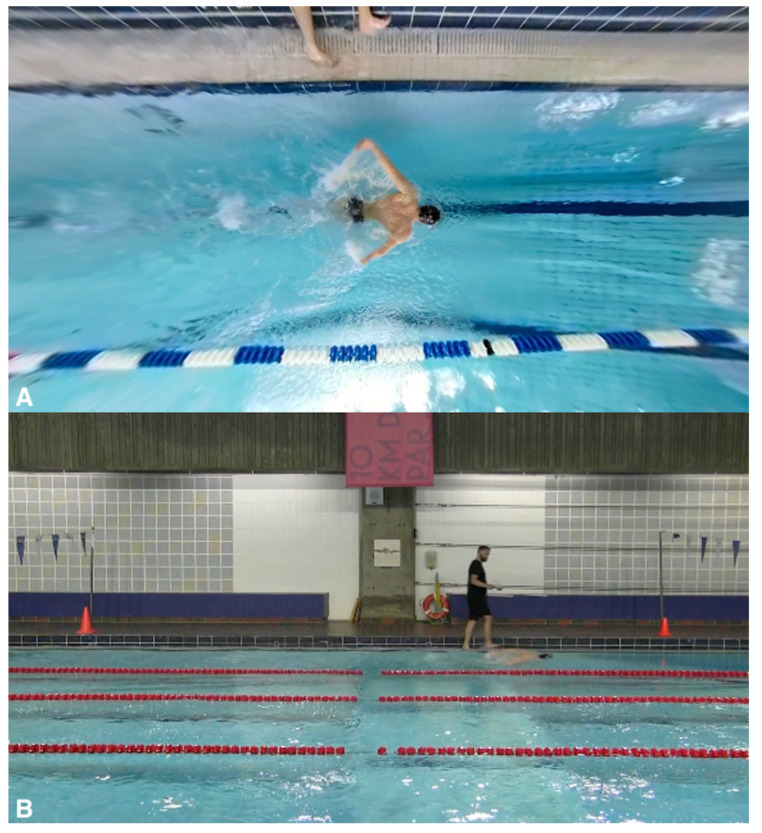
The experimental setup for the data collection shows (**A**) the above camera view and (**B**) the stationary camera view.

A 360° video camera (60 Hz, Fusion, GoPro Inc., San Mateo, CA, USA) was used as a reference to record the timing of stroke cycles. A second stationary camera (30 Hz, Vixia HR G10, Canon Inc., Tokyo, Japan) was positioned to capture a 10 m section of the pool for measurement of DPS. See Figure 2 for a visual representation of the experimental set-up.

### 2.3. Protocol

The athlete was instrumented during a regular season training session. A 15 min warm-up was completed prior to the protocol, individualized for the athlete, and prescribed by the head coach. The protocol required that athletes perform a 4 × 50 m protocol (the sequence being 50 m of butterfly, 50 m of backstroke, 50 m of breaststroke, and lastly, 50 m of freestyle). Each athlete was asked to swim at a rate of perceived exertion of 7/10 on the Borg CR-10 scale [26]. Each athlete rested for a minimum of 1 min between each 50 m trial. Each 50 m trial was initiated from a dive start at the end of the pool where the previous 50 m trial was terminated. The stroke types performed by the athlete were prescribed by the head coach of the swimming team based on their capabilities, availability during training, and fatigue/injury status. A total of 29 individual stroke trials were recorded (freestyle = 8, backstroke = 7, breaststroke = 8, butterfly = 6).

Video clips were collected using a video camera attached to an extendable pole positioned directly above the athlete, allowing for a ‘birds-eye’ view of each swimming trial (Figure 2A). An operator carried the pole along the side of the pool so that the camera remained above the athlete throughout the trials. To allow for optimal camera positioning, all trials were performed in the lane closest to the poolside. A second stationary camera recorded each swim trial over the middle 10 m section of the pool. IMU and video data were synchronized by tapping the IMU three times in clear view of both cameras.

### 2.4. Data Processing

#### 2.4.1. IMU Processing

All data were processed with custom-made software (Matlab 2023a, Mathworks, Natick, MA, USA). Instantaneous velocity and position were calculated as described previously [14]. Five trial events were manually defined by a trained operator: (1) dive start, (2) dive end, (3) start of underwater kicks, (4) end of underwater kicks/start of swim, and (5) end of swim.

Stroke cycles were identified using a zero-crossing algorithm [18] applied to the medio-lateral (freestyle and backstroke) or forward-backward (butterfly and breaststroke) instantaneous velocity data. Details of the calculations for each stroke parameter from the IMU data are presented in Table 2.

#### 2.4.2. Video Processing

Video data from the camera positioned above the athlete (see Figure 2A) were rendered using Fusion Studio (Version 1.2, GoPro Inc., San Mateo, CA, USA). Video data were converted from 360° to 2-D using GoPro Player’s “Overcapture” feature (Version 2.1.6, GoPro Inc., San Mateo, CA, USA). Two-dimensional videos were analyzed in Dartfish (Version 8, Dartfish, Fribourg, Switzerland), with stroke cycle start points tagged. Definitions of the video-based start point of strokes are presented in Table 3.

Stroke cycle start points were tagged at two separate times by the same trained operator, rendering two time series extracted from each video sequence, then used to assess the intra-rater reliability of the method. Pearson’s R correlations between the two video tagging datasets showed extremely strong correlations (r = 0.999, n = 1042), confirming the relative reliability of the large effect [27]. A root mean square error (RMSE) of 64 ms (df = 1040, *p* < 0.001), or roughly 4 video frames at 60 Hz, between the datasets were observed. Therefore, given this evidence for the very high intra-rater reliability of the video tagging method, the means of the two intra-rater video-derived stroke cycle start timings were then used as a comparison to the IMU-derived stroke cycle start points.

Images from the stationary video camera were analyzed in Kinovea (Version 0.9.5, Kinovea open-source project, www.kinovea.org, accessed on 1 September 2023). Above-water and stationary camera video data were synchronized using the first frame of toe-off from the above-swimmer camera operator after the swimmer had passed the red cones (positioned to indicate the stationary camera capture space). This method of synchronization was used as the camera views of the operator were not impeded by water turbulence or splashes. The 10-meter capture space was calibrated using Kinovea’s ‘perspective grid’ function (calibrated as the 2.5 m lane width × the 10 m lane length between the red cones). The head trajectory of the athlete was tracked through the capture space. DPS was calculated as the displacement in meters of the athlete from the start to the end of each stroke cycle using the stationary video camera. For freestyle and backstroke, distances per stroke were calculated as the displacement between stroke cycle start points for each arm independently. Details of the calculations for each stroke parameter from the video data are presented in Table 2.

### 2.5. Statistical Analyses

Agreement between video and IMU-derived SC, stroke durations, ISR, DPS, and lap times were assessed using Bland–Altman analyses [28]. Bland–Altman plots, ninety-five percent (95%) limits of agreement (LoA), and RMSE were calculated using Microsoft Excel (Version 16.76, Microsoft Corporation, Redmond, WA, USA). 95% LoA was defined as the mean difference between video and IMU ± 1.96 standard deviations (SD). All further statistical analyses were completed for each type of stroke using R (Version 4.2.1, R Core Team, 2018). Scattering of data around the bias, skewness, and kurtosis confirmed a normal data distribution [15], using the R “moments” package (Version 0.14.1, Komsta & Novomestky, 2022). Single measures of intraclass correlation coefficients (ICC) in a two-way model on absolute agreement (ICC 2, 1) were computed for all outcome variables [29] using the R “psych” package (Version 2.3.6, Revelle, 2023). ICC less than 0.40 was considered poor; 0.40 to 0.59 was fair; 0.60 to 0.74 was good; and 0.75 to 1.00 was excellent [25]. The standard error of measurement (SEM) was calculated as SD × √(1 − ICC), using base R. Mean Absolute Percentage Error (MAPE) was calculated for all outcome variables using the R “MLmetrics” package (Version 1.1.1, Yan, 2016). The coefficient of variation (CV) was calculated for all IMU and video-derived outcomes using base R.

## 3. Results

### 3.1. Stroke Count

The results of the Bland–Altman analyses and agreement analyses for stroke counts are detailed in Table 4. The Bland–Altman analyses showed an overall bias of 0.13 strokes (95% CI [−0.83; 1.07]). The RMSE was 0.49 strokes. Agreement analyses revealed an ICC of 0.97 (95% CI [0.93; 0.98]). The MAPE was 0.66%. Similar CVs between both data sources were observed.

### 3.2. Stroke Duration

**Figure 3:**
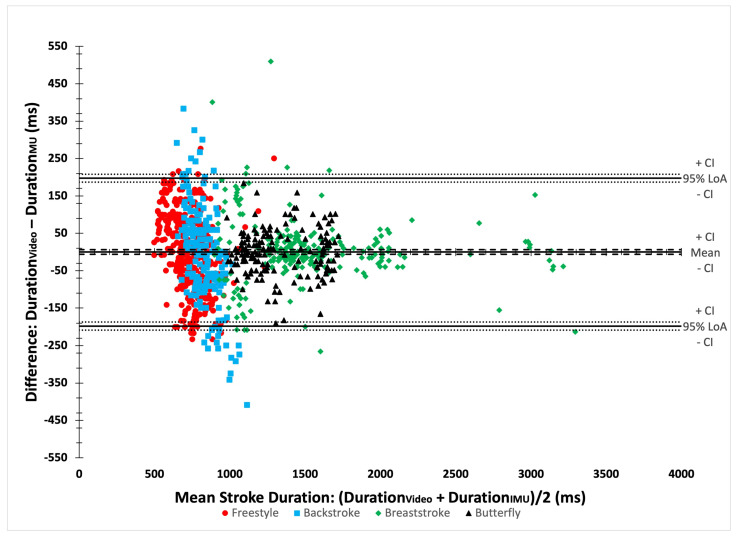
A Bland–Altman plot comparing video and IMU stroke durations.

The results of the Bland–Altman analyses (Figure 3) and agreement analyses for stroke durations are detailed in Table 5. The Bland–Altman analyses showed an overall bias of −0.15 ms (95% CI [−6.37; 6.07]). The RMSE was 100.81 ms for all strokes. Agreement analyses revealed an ICC of 0.97 (95% CI [0.97; 0.98]). The MAPE was 8.78%. Similar CVs between both data sources were observed.

### 3.3. Instantaneous Stroke Rate (ISR)

**Figure 4:**
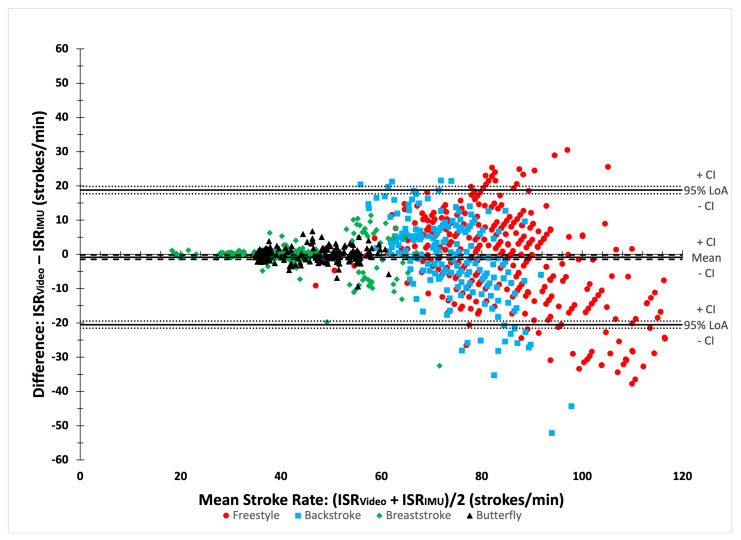
A Bland–Altman plot comparing video and IMU ISR.

The results of the Bland–Altman analyses (Figure 4) and agreement analyses for ISR are detailed in Table 6. The Bland–Altman analyses showed an overall bias of −0.84 strokes per min (95% CI [−1.46; −0.22]). The RMSE was 10.05 strokes per min. Agreement analyses revealed an ICC of 0.90 (95% CI [0.88; 0.91]). The MAPE was 8.97%. CV were similar between both data sources for breaststroke and butterfly but differed in the bilateral strokes.

### 3.4. Distance Per Stroke (DPS)

**Figure 5:**
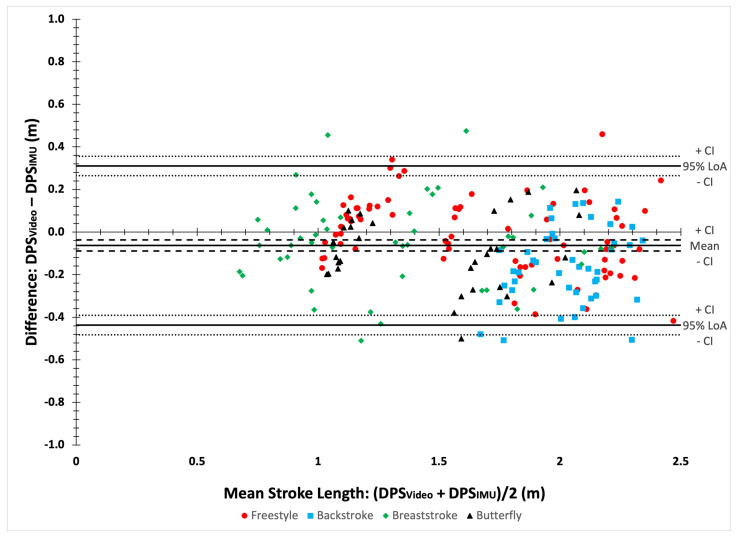
A Bland–Altman plot comparing video and IMU distance per stroke (DPS).

The results of the Bland–Altman analyses (Figure 5) and agreement analyses are detailed in Table 7. The Bland–Altman analyses showed an overall bias of −0.06 m (95% CI [−0.09; −0.04]). The RMSE was 0.20 m for all strokes. Agreement analyses revealed an ICC of 0.91 (95% CI [0.88; 0.93]). The MAPE was 10.78%. CV showed stroke-specific differences between data sources.

### 3.5. Lap Time

The results of the Bland–Altman analyses and agreement analyses are detailed in Table 8. The Bland–Altman analyses showed an overall bias of 0.04 s (95% CI [−0.09; 0.02]). The RMSE was 0.15 s for all strokes. Agreement analyses revealed an ICC of 1 (95% CI [1; 1]). The MAPE was 0.32%. Similar CVs between both data sources were observed.

## 4. Discussion

In this study, we showed how a single IMU can be used to obtain in-field assessments of swim stroke variables that scientists and coaches can relate to the biomechanical risk of fatigue and injury during para-swimming. Results from the present study highlight overall good to excellent validity in the IMU system, with some discrepancies in specific parameters and strokes. The current IMU system can accurately estimate stroke count and lap times with a high level of confidence. Swim cycle stroke-to-stroke parameters (stroke duration, instantaneous stroke rate, and distance per stroke) showed the strongest validity for butterfly and breaststroke; analyses of freestyle showed lesser validity for cycle-to-cycle timing parameters (stroke duration and instantaneous stroke rate); and backstroke performed poorly for all cycle-to-cycle stroke parameters.

### 4.1. Stroke Count

Results from the present study show similar magnitudes of error comparing the IMU to video-derived stroke counts, falling between ± 1 [30,31] and ± 2 strokes of error for freestyle swimming [11,32,33]. This is in agreement with previous findings of errors of less than 1 stroke when comparing a sacrum-worn IMU to video-derived stroke counts for butterflies [34]. Commercially available smart watch devices that estimate stroke count have reported MAPEs of 6.2–9.3% (Apple Watch S2) and 6.8–17.6% (Garmin Finex 3HR) [35]. Additionally, the head-worn TritonWear unit showed MAPEs of 0%, 7.1%, 2.4%, and 4.9% for butterfly, backstroke, breaststroke, and freestyle, respectively, higher than the results of the present study [36]. Together, this highlights the excellent validity of the current system to estimate stroke count, performing similarly to this research consensus and outperforming commercial devices. As such, the current system is a valid tool to monitor the number of stroke cycles performed by athletes while training, as highlighted by the low SEM for all strokes, aiding workload monitoring practices. One possible use case for this tool would be to track the number of stroke cycles performed per week to quantify the reintroduction of athletes to in-season training loads following an injury.

### 4.2. Instantaneous Stroke Rate (ISR)

Although previous studies had reported on instantaneous stroke rates derived from sacrum-worn IMUs [18], validations were only performed for freestyle in able-bodied swimmers. The present study validated the instantaneous stroke rate for all four strokes in an elite para-swimming population. Our results on instantaneous stroke rate errors for butterfly and breaststroke are similar to previous lap averaged stroke rate errors in the literature [30], but we report higher errors for freestyle and backstroke. A possible explanation for this is the medio-lateral zero-crossing stroke detection algorithm, which assumes body roll and associated lateral accelerations while swimming oscillates cyclically [18]. In the current study, the highest errors in backstroke and freestyle were observed for athletes 1, 3, and 4 (Table 1). While each of these athletes has differing impairments, it is proposed that a common kinematic outcome of these impairments is irregularity in their lateral accelerations, since the algorithm uses this cyclical motion to detect stroke cycles. To directly compare against the literature, we estimated the lap averaged stroke rate from the collected data and observed MAPEs of 0.8%, 1.6%, 4.9%, and 2.2% for butterfly, backstroke, breaststroke, and freestyle, respectively. These values are similar in magnitude to the previous literature [36]. With the validated tool, instantaneous stroke rate can be used to determine an athlete’s ability to adequately deal with the demands of training and competition. The point at which instantaneous stroke rate reduces significantly during a constant-intensity training session can be inferred as the point where the negative effects of fatigue cannot be mitigated by an athlete. As such, instantaneous stroke rate can be used in monitoring practices to determine an athlete’s endurance capacity in specific events. This can allow for targeted interventions, such as strength and conditioning, to improve an athlete’s endurance capacity, specifically when they fatigue. However, athlete impairment and stroke type should be considered when determining the thresholds used to identify temporal changes in instantaneous stroke rate. This is due to the variation in sensitivity of this metric, as highlighted by the differing SEMs by stroke type.

### 4.3. Distance per Stroke (DPS)

A previous study also presented the distance per stroke derived from an IMU placed on the sacrum [19]. However, no study to date has directly validated IMU-derived distance per stroke against gold standard video. To compare our results directly to those of the literature, we estimated lap averaged stroke length from the collected data and observed MAPEs of 5.7%, 6.8%, 10.4%, and 6.4% for butterfly, backstroke, breaststroke, and freestyle, respectively, similar in magnitude to the previous literature [36]. For backstroke and freestyle, the largest errors between video and IMU-derived distance per stroke were observed for athletes 3, 4, and 8 (Table 1). As with ISR, the common kinematic outcome of the varying impairments between the abovementioned athletes is irregularities in lateral accelerations. Once again, this may reduce the ability of the zero-crossing algorithm to detect stroke cycles, introducing errors into the distance per stroke estimations. However, SEMs for each stroke type ranged from 0.12 m to 0.17 m, highlighting small levels of error and minimal variation between stroke types. As such, distance per stroke, as validated in the present study, can provide novel information to coaches, athletes, and support staff about relative changes in stroke length within one lap. This information may provide time-based indices of when an athlete cannot mitigate the negative effects of fatigue while swimming. This metric can be used in combination with instantaneous stroke rate to better determine when and how fatigue affects an athlete during their training and competitions. This could allow for specific interventions to target the kinematic changes seen in cycle-to-cycle stroke parameters.

### 4.4. Lap Time

In previous literature comparing IMU (or accelerometer) to video-derived lap times in 100 m freestyle, the majority of lap time errors fell within ± 0.5 [32] to ± 1 s [33]. In the present study, a 95% LoA of [−0.35; 0.16] seconds were reported, suggesting the majority of the dataset for freestyle had an error of ± 0.5 s. The present study reported lower MAPEs in comparison to the head-worn TritonWear unit [36]. It should be noted that the present study used a trained operator to manually define the start and end points of the swim trial as a step in the data processing workflow, whereas the literature reports times based on automated start and end points. While the reported values in the literature are higher than those of the present study, they represent automatic solutions that could be easier to incorporate as part of a regular performance monitoring workflow. However, validation of this parameter allows lap times to be monitored longitudinally across specific training sets over a season. As such, progression and regression in swimming performance can be determined. This may allow for the identification of overtraining or maladaptation to training, a current practice completed manually by coaches via stopwatches that can be improved via longitudinal parameter monitoring.

### 4.5. Limitations and Future Work

In the present study, some limitations should be highlighted. First, the sample size (n = 8) is small. However, this sample represents the entire population of para-swimming athletes training with the studied group, meaning the results are representative of the in-tended end user [37]. Additionally, as the athletes with a range of impairments performed the four swimming strokes, a high level of inter-subject variability were represented in the present dataset (see Table 1). Secondly, tagging swim stroke events using video was sometimes unclear due to water turbulence, which may introduce some errors into the validation analyses. However, high intra-rater reliability was confirmed, and mean values were used for the statistical analyses to minimize operator error introduced into the validation analyses. Video-derived distance per stroke was calculated following the trajectory of the swimmer’s head, while the IMU-derived parameter is estimated from sacrum displacement. This introduces some errors into the validation analyses. Lastly, the underlying calculations used to estimate distance per stroke assumed that the IMU traveled 50 m per lap. The IMU moved from the dive start position outside of the pool to a position corresponding to the moment when the athletes touched the opposite end of the pool. The actual distance traveled by the IMU includes a change in vertical displacement in the dive phase until the beginning of the swimming phase at the surface of the water, plus the swimming phase distance, minus the distance between the hand touching the wall and the IMU. Future work should consider (1) alternate algorithms to detect stroke cycles for freestyle and backstroke in para-swimmers and (2) the validity of automated lap time estimations in elite para-swimmers.

## 5. Conclusions

The present study shows excellent validity between the IMU and video-based systems for stroke count and lap time estimation in para-swimmers, matching or exceeding the current state of the art in the literature, and doing so for the first time for all strokes. The system also shows good to excellent validity in stroke duration, ISR, and DPS for all strokes except backstroke. As such, the current system can be used as a valid tool to monitor the number of movement repetitions performed over trials, training sessions, and weeks, allowing for in-pool workload monitoring specific to swim strokes. Additionally, ISR and DPS can be used together to determine when and how fatigue affects an athlete’s kinematic patterns in a competitive event or training session. This can ultimately allow for interventions to be personalized to the individual to better prepare them for the demands of their performances and reduce injury risk. However, special attention should be given when using this tool with athletes that have impairments that affect the regularity of lateral accelerations in freestyle and backstroke.

## Figures and Tables

**Table 1:** Details of disabilities, para-swimming classes of the athletes, and strokes performed.

Athlete ID	Disabilities	Classes	Swimming Strokes
1	Right femoral-fibula-ulnar syndrome; Dysmeliac right upper limb	S8, SB7, SM8	FY, BK, BR, FR
2	Intellectual Impairment	S14, SB14, SM14	FY, BK, BR, FR
3	Dysmelia, congenital left hand amputee	S10, SB9, SM10	FY, BK, BR, FR
4	Cerebral palsy	S9, SB8, SM9	FY, BK, BR, FR
5	Pseudoachondroplasia	S5, SB5, SM5	BR, FR
6	Achondroplasia dwarfism	S6, SB6, SM6	BK, BR, FR
7	Stroke (post-bleeding aneurysm)	S7, SB6, SM7	FY, BK, BR, FR
8	Congenital impaired strength loss at the left hip, knee, and ankle with associated foot deformity	SB9	FY, BK, BR, FR

World Para Swimming classifies freestyle, butterfly, and backstroke events as S, breaststroke as SB, and individual medley as SM. A lower number indicates a more severe activity limitation. Physical impairment numbers range from 1 to 10, and intellectual impairments are classified as 14. FY = Butterfly, BK = Backstroke, BR = Breaststroke, and FR = Freestyle.

**Table 2:** Description of IMU and video calculations for each stroke parameter.

Stroke Parameter	Unit	IMU Description	Video Camera	Video Description
Stroke count (SC)	count	Sum of detected stroke cycles per lap.	Above swimmer	Sum of tagged stroke cycles per lap.
Stroke duration	ms	Time difference between the n^th^ and n^th^ + 1 stroke cycle start points.	Above swimmer	Time difference between the n^th^ and n^th^ + 1 stroke cycle starting points.
Instantaneous stroke rate	Strokes/min	Estimated rate of strokes per minute (60/duration of a given stroke in seconds).	Above swimmer	Stroke duration (s) divided by 60 s.
Distance per stroke (DPS)	m	Displacement between the n^th^ and n^th^ + 1 stroke cycle start points.	Stationary and above swimmer	Displacement (stationary camera) between the n^th^ and n^th^ + 1 stroke cycle starting points (taken from the above swimmer camera).
Lap Time	s	Time difference between the 1st and 5th manually defined swim events.	Above swimmer	Time difference between the first frame where the athlete initiated the dive and the first frame where the athlete touched the wall.

**Table 3:** Definitions of the video start points of the stroke cycle for the four swimming styles.

Stroke Style	Definition of the Start of the Stroke Cycle
Freestyle	The start of torso rotation as the arm recovery phase is ending.
Backstroke	The start of the arm pull phase after the catch phase has finished.
Breaststroke	The start of the arm-pull phase after the sculling-out movement has finished.
Butterfly	The start of the arm pull phase after the catch phase has finished.

**Table 4:** Bland–Altman and agreement results for stroke count.

	N	RMSE (Strokes)	Count Mean ± SD	Bias (Strokes) [95% CI]	95% Limits of Agreement [95% CI]		ICC [95% CI]	SEM (Strokes)	CV		MAPE (%)
					Lower Limit (Strokes)	Upper Limit (Strokes)			IMU	Video	
Overall	28	0.49	27.54 ± 9.73	0.13 [−0.05; 0.30]	−0.82 [1.13; 0.51]	1.07 [0.76; 1.38]	0.97 [0.93; 0.98]	1.72	35.37	35.28	0.66
Freestyle	8	0.59	25.59 ± 9.49	0.31 [−0.06; 0.68]	−0.73 [−1.36; −0.09]	1.35 [0.72; 1.99]	1 [0.99; 1]	0	36.58	37.58	0.91
Backstroke	7	0.38	24.14 ± 7.40	0.29 [0.09; 0.48]	−0.24 [−0.58; 0.10]	0.81 [0.47; 1.15]	1 [0.99; 1]	0	30.54	30.81	1.10
Breaststroke	8	0.66	32.07 ± 12.54	−0.14 [−0.65; 0.37]	−1.50 [−2.38; −0.61]	1.21 [0.32; 2.10]	1 [0.99; 1]	0	39.25	38.99	1.71
Butterfly	6	0	28.83 ± 8.80	0 [0; 0]	0 [0; 0]	0 [0; 0]	1 [1; 1]	0	30.51	30.51	0

**Table 5:** Bland–Altman and agreement results for stroke duration.

	N	RMSE (ms)	Duration (ms) Mean ± SD	Bias (ms) [95% CI]	95% Limits of Agreement [95% CI]		ICC [95% CI]	SEM (ms)	CV		MAPE (%)
					Lower Limit (ms)	Upper Limit (ms)			IMU	Video	
Overall	1011	100.81	1042 ± 430	−0.15 [−6.37; 6.07]	−197.83 [−187.06; −208.59]	197.53 [186.76; 208.29]	0.97 [0.97; 0.98]	75.05	41.93	41.25	8.78
Freestyle	399	103.05	735 ± 114	0.17 [−9.95; 10.30]	−202.05 [−219.59; −184.52]	202.40 [184.86; 219.94]	0.66 [0.60; 0.71]	72.81	18.87	14.84	11.78
Backstroke	200	132.03	1605 ± 831	−4.51 [−22.84; 13.83]	−263.79 [−295.55; −231.28]	254.78 [223.02; 286.53]	0.30 [0.17; 0.42]	93.15	16.78	9.00	12.48
Breaststroke	245	87.97	1322 ± 224	3.47 [−7.56; 14.51]	−166.17 [−184.95; −147.40]	173.12 [154.35; 191.89]	0.98 [0.97; 0.99]	67.32	31.52	30.95	4.34
Butterfly	167	62.58	826 ± 89	−1.01 [−10.53; 8.51]	−124.01 [−140.50; −107.53]	121.99 [105.51; 138.48]	0.96 [0.95; 0.97]	45.04	17.07	17.18	3.71

**Table 6:** Bland–Altman and agreement results for ISR.

	N	RMSE (Strokes/min)	ISR (Strokes/min) Mean ± SD	Bias (Strokes/min) [95% CI]	95% Limits of Agreement [95% CI]		ICC [95% CI]	SEM (Strokes/min)	CV		MAPE (%)
					Lower Limit (Strokes/min)	Upper Limit (Strokes/min)			IMU	Video	
Overall	1011	10.05	65.74 ± 21.27	−0.84 [−1.46; −0.22]	−20.48 [−21.55; −19.41]	18.80 [17.73; 18.80]	0.90 [0.88; 0.91]	6.98	35.29	32.00	8.97
Freestyle	399	12.90	83.91 ± 13.03	−1.45 [−2.71; 0.19]	−26.61 [−28.79; −24.61]	23.71 [21.53; 25.89]	0.61 [0.54; 0.67]	9.08	20.03	13.95	12.02
Backstroke	200	12.31	73.94 ± 8.01	−1.06 [−2.77; 0.64]	−25.17 [−28.12; −22.16]	23.04 [20.09; 26.00]	0.26 [0.12; 0.38]	8.69	16.97	9.06	12.84
Breaststroke	245	4.30	41.95 ± 11.45	−0.26 [−0.81; 0.28]	−8.78 [−9.72; −7.83]	8.25 [7.31; 9.19]	0.92 [0.90; 0.94]	3.29	28.29	27.21	4.52
Butterfly	167	2.25	46.65 ± 7.58	0.05 [−0.29; 0.39]	−4.38 [−4.97; −3.79]	4.47 [3.88; 5.07]	0.96 [0.94; 0.97]	1.53	16.41	16.48	3.70

**Table 7:** Bland–Altman and agreement results for distance per stroke.

	N	RMSE (m)	DPS (m) Mean ± SD	Bias (m) [95% CI]	95% Limits of Agreement [95% CI]		ICC [95% CI]	SEM (m)	CV		MAPE (%)
					Lower Limit (m)	Upper Limit (m)			IMU	Video	
Overall	198	0.20	1.63 ± 0.47	−0.06 [−0.09; −0.04]	−0.44 [−0.48; −0.39]	0.31 [0.26; 0.36]	0.91 [0.88; 0.93]	0.14	29.61	28.54	10.78
Freestyle	75	0.17	1.68 ± 0.46	−0.01 [−0.05; 0.03]	−0.35 [−0.42; −0.28]	0.33 [0.27; 0.40]	0.93 [0.89; 0.96]	0.12	29.41	26.30	8.54
Backstroke	43	0.24	2.03 ± 0.18	−0.16 [−0.21; −0.11]	−0.50 [−0.59; −0.41]	0.18 [0.09; 0.18]	0.39 [0.11; 0.62]	0.17	8.46	11.13	10.55
Breaststroke	48	0.21	1.31 ± 0.44	−0.04 [−0.10; 0.01]	−0.45 [−0.55; 0.35]	0.36 [0.26; 0.47]	0.89 [0.81; 0.94]	0.15	34.13	34.67	14.15
Butterfly	32	0.19	1.48 ± 0.36	−0.09 [−0.15; −0.03]	−0.42 [−0.53; −0.32]	0.24 [0.14; 0.34]	0.87 [0.75; 0.93]	0.13	23.93	25.56	11.31

**Table 8:** Bland–Altman and agreement results for lap time.

	N	RMSE (s)	Lap Time (s) Mean ± SD	Bias (s) [95% CI]	95% Limits of Agreement [95% CI]		ICC [95% CI]	SEM (s)	CV		MAPE (%)
					Lower Limit (s)	Upper Limit (s)			IMU	Video	
Overall	30	0.15	48.29 ± 16.35	0.04 [−0.09; 0.02]	−0.34 [−0.43; −0.24]	0.26 [0.17; 0.35]	1 [1; 1]	0	33.87	33.86	0.32
Freestyle	8	0.16	42.04 ± 13.43	−0.10 [−0.19; 0.01]	−0.35 [−0.51; −0.20]	0.16 [0.00; 0.32]	1 [1; 1]	0	31.84	32.05	0.39
Backstroke	6	0.14	46.11 ± 8.50	−0.03 [−0.15; 0.09]	−0.32 [−0.52; 0.12]	0.26 [0.05; 0.46]	1 [1; 1]	0	18.50	18.37	0.28
Breaststroke	8	0.12	57.16 ± 19.37	−0.01 [−0.10; 0.08]	−0.26 [−0.41; 0.11]	0.23 [0.08; 0.38]	1 [1; 1]	0	33.91	33.87	0.17
Butterfly	8	0.19	47.31 ± 19.00	−0.01 [−0.15; 0.13]	−0.41 [−0.65; −0.16]	0.39 [0.14; 0.63]	1 [1; 1]	0	40.25	40.07	0.42

## Data Availability

The data presented in this study are available on request from the corresponding author. The data are not publicly available due to the confidentiality of the sampled participants.
